# Variations in Delivery and Exercise Content of Physical Therapy Rehabilitation Following Total Knee Replacement Surgery: A Cross-Sectional Observation Study

**DOI:** 10.4172/2329-9096.S5-002

**Published:** 2014-04-22

**Authors:** Carol A. Oatis, Wenjun Li, Jessica M. DiRusso, Mindy J. Hoover, Katherine K. Johnston, Monika K. Butz, Amy L. Phillips, Kimberly M. Nanovic, Elizabeth C. Cummings, Milagros C. Rosal, David C. Ayers, Patricia D. Franklin

**Affiliations:** 1 Department of Physical Therapy, Arcadia University, 450 S. Easton Road, Glenside, PA 19038, USA; 2 Department of Orthopedics and Physical Rehabilitation, University of Massachusetts Medical School, 55 Lake Avenue North, Worcester, MA 01655, USA; 3 Department of Medicine, Division of Preventive and Behavioral Medicine, University of Massachusetts Medical School, 55 Lake Avenue North, Worcester, MA 01655, USA; 4 The Queens Medical Center, 1301 Punchbowl St, Honolulu, HI 96813, USA; 5 The Physical Therapy Associates of Myerstown, 11 E Lincoln Ave, Myerstown, PA 17067, USA; 6 Sports and More Physical Therapy, Inc., 8300 Health Park, Suite 127, Raleigh, NC 27615, USA; 7 Drayer PT Institute, 998 Hospitality Way, Aberdeen, MD 21001, USA; 8 UPMC Shadyside, 5230 Centre Ave, Pittsburgh, PA 15232, USA; 9 St. Luke’s University Health Network, 801 Ostrum Street, Bethlehem, PA 18015, USA; 10 HealthEast Optimum Rehabilitation, Midway Clinic, 1390 University Avenue West Saint Paul, Minnesota 55104, USA

**Keywords:** Physical therapy, Rehabilitation, Surgery, Knee replacement

## Abstract

**Objective:**

Prevalence of total knee replacement (TKR) is large and growing but functional outcomes are variable. Physical therapy (PT) is integral to functional recovery following TKR but little is known about the quantity or content of PT delivered. Purposes of this study were to describe the amount and exercise content of PT provided in the terminal episode of PT care following TKR and to examine factors associated with utilization and content.

**Methods:**

Subjects included participants in a clinical trial of behavioral interventions for patients undergoing primary unilateral TKR who had completed the 6-month study evaluation. PT records were requested from 142 consecutive participants who had completed their post-TKR rehabilitation, 102 in/out patient care, and 40 in homecare. Information on utilization and exercises was extracted from a retrospective review of the PT records.

**Results:**

We received 90 (88%) outpatient and 27 (68%) homecare PT records. Records showed variability in timing, amount and content of PT. Patients receiving outpatient PT had more visits and remained in PT longer (p<0.001). Exercises known in the TKR literature were utilized more frequently in the outpatient setting (p=0.001) than in home care. Records from both settings had limited documentation of strengthening progression.

**Conclusions:**

The study reveals considerable variability in timing, utilization and exercise content of PT following TKR and suggests sub-optimal exercise for strengthening. While methods we employed document variability, improved systematic PT documentation and in-depth research are needed to identify optimal timing, utilization and content of PT following TKR.

## Introduction

Over 650,000 total knee replacements (TKR) were performed in the US in 2010. That number is projected to grow to almost 3.5 million by 2030 [1,2]. The greatest growth in the TKR population is in those under 65 years [3]. Although this procedure is highly successful in relieving pain, Franklin et al report that over 36% of patients report little or no functional improvement one year post TKR [4]. Rehabilitation is an integral and routine intervention following TKR directed toward restoring function, but little is known about the actual amount or content of rehabilitation services provided to patients following TKR. DeJong and colleagues examined the total amount of PT provided to post-TKR patients admitted to either a skilled nursing or inpatient rehabilitation facility. Patients were followed for 7.5 months following discharge from acute care. On average patients had a total of 19 PT visits, utilization varied considerably [5]. No explanation was provided about how visits were distributed among various treatment settings, including homecare and outpatient care. Fritz et al reported that Medicare patients seen in outpatient PT within one integrated health care system, had an average of 11.6 visits following TKR [6]. The average age of the subjects in both of these studies was over 70 years, yet 40% of current TKR patients are under 65 years of age.

Little information is available describing content of PT services following TKR although there is consensus for the need to increase strength and knee range of motion [7,8]. DeJong et al describe the general content of PT treatments provided to patients in skilled nursing and inpatient rehabilitation facilities in patients following TKR [9]. Although the most common treatment was therapeutic exercise for strengthening and stretching, at least half of treatment time was devoted to other interventions including bed mobility, transfer and gait training, and pain management. No details were provided about the kind or intensity of therapeutic exercises. These interventions were provided typically within the first two weeks following discharge from acute care with little data describing subsequent rehabilitation interventions.

To our knowledge, there are no published studies detailing PT interventions patients typically receive following the acute phase of recovery. However evidence exists supporting the role of vigorous exercise and quadriceps strengthening to improve functional outcomes following TKR. Studies demonstrate that functional, weight-bearing, and progressive quadriceps strengthening exercises lead to improved strength and function [10–12].

Our study’s primary purpose was to describe the timing, amount and exercise content of PT received by patients in their terminal episode of post-TKR care. The terminal episode of care was defined as the set of PT services that completed a patient’s rehabilitation following TKR. We focused our investigation on PT services provided either at home or an outpatient facility following any inpatient rehabilitation services. Outpatient and home care PT has become increasingly important because access to inpatient rehabilitation following TKR has been limited in the last decade [13] and length of stay in acute care has steadily decreased in the last two decades, averaging approximately 4 days [14]. Finally, studies demonstrate that quadriceps muscle strength decreases immediately following surgery and is less than pre-operative levels at four weeks post- TKR [15]. Consequently the most vigorous rehabilitation is likely to occur in the final rehabilitation setting. These factors also explain why we focused on the exercise interventions during rehabilitation. Additional physical modalities may be used to reduce pain and swelling. However exercise is typically the focus of intervention in the post-acute phase.

We also analyzed differences in the amount and content of the terminal episode of PT delivered at home compared to outpatient sites, and identified patient factors associated with such differences.

## Methods

This is a cross-sectional observation study of standard PT administered to patients following TKR. Data were obtained from PT records of individuals enrolled in the Joint Action study at the University of Massachusetts Medical Center between 2008 and 2011. The methods of the clinical trial are discussed elsewhere [16]. This study was approved by the Institutional Review Board of the Office of Human Subjects at the University of Massachusetts Medical School and by the Institutional Review Board of the Committee for the Protection of Research Subjects at Arcadia University.

All participants in the study received usual operative and rehabilitation care according to the patient’s and clinician’s preferences. Participants were consecutively enrolled from all individuals 21 years of age or older scheduled for primary TKR surgery at the Arthritis and Joint Replacement Center. More than 95% of eligible patients enrolled. Exclusions included inflammatory arthritis, TKR due to fracture, malignancy, infection, or failure of a previous arthroplasty; inability to provide informed consent due to dementia or cognitive impairment; co-existing conditions preventing functional improvement from surgery; emergently scheduled surgery; simultaneous bi-lateral TKR; or terminal illness with life expectancy less than one year. All participants signed releases to allow study investigators to review their health records during the study period.

Baseline variables included demographic and psychosocial characteristics: age, gender, insurance status, physical co-morbid conditions [17], and body mass index (BMI). Health status and function were assessed by the SF-36 and the joint specific Western Ontario and McMaster Universities Osteoarthritis Index (WOMAC) [18,19].

All study participants were treated by three dedicated arthroplasty surgeons at one high volume center using a consistent peri-operative protocol for inpatient care. Patients chose their PT provider and received usual care PT as prescribed by their healthcare provider. At their 6-month study assessment, participants identified the facilities where they had received PT following surgery and stated whether they had completed their course of PT. Subjects reported their insurance co-payments for PT. We requested outpatient PT records from the first 102 subjects who had completed their six-month study assessment and completed outpatient PT for their study knee. Additionally we requested homecare PT records of the first 40 subjects who had completed their six-month study assessment and completed rehabilitation of their study knee in homecare with no additional outpatient PT.

We reviewed all PT records to determine the timing and duration of PT and exercise interventions provided at each visit. Utilization variables included total number of PT visits, days from TKR surgery to onset of PT (either outpatient or homecare), days from TKR surgery to discharge from PT, and days from initial PT evaluation to discharge.

Because evidence supports the use of progressive strengthening and dynamic exercise to improve function following TKR, we reviewed the records to identify documentation of progressive strengthening of the quadriceps and use of any exercises supported in the literature. Progressive strengthening of the quadriceps was defined as an increase in resistance in quadriceps exercise such as a change in the color of a resistance band or an increase in difficulty in an exercise, for example progressing from a quadriceps set to a straight leg raise. We selected exercises identified in published treatment guidelines [20] or included in exercise regimens from clinical trials found to improve functional outcomes following TKR [11,12,21] then reviewed records to determine which of these exercises were documented in the episode of care. Investigators with advanced PT training conducted data extraction and were trained to ensure that each exercise identified in a record was documented and classified in the same way. Two investigators independently reviewed each record. Differences between extractors were resolved through discussion and if necessary adjudicated by the lead PT investigator (CAO).

### Data analysis

We used descriptive statistics to describe PT utilization and content for subjects treated in either outpatient or homecare PT. Analysis of variance and Wilcoxon rank sum test were used to identify associations between baseline characteristics and utilization variables. Logistic regression models were used to identify baseline characteristics predictive of use of homecare as the terminal rehabilitation site. Tests of equal proportion and medians were used to compare intervention patterns between treatment settings. All statistical analyses were performed using Stata Statistical Software: Release 12. StataCorp. 2011, StataCorp LP, College Station, TX.

## Results

**Figure 1:**
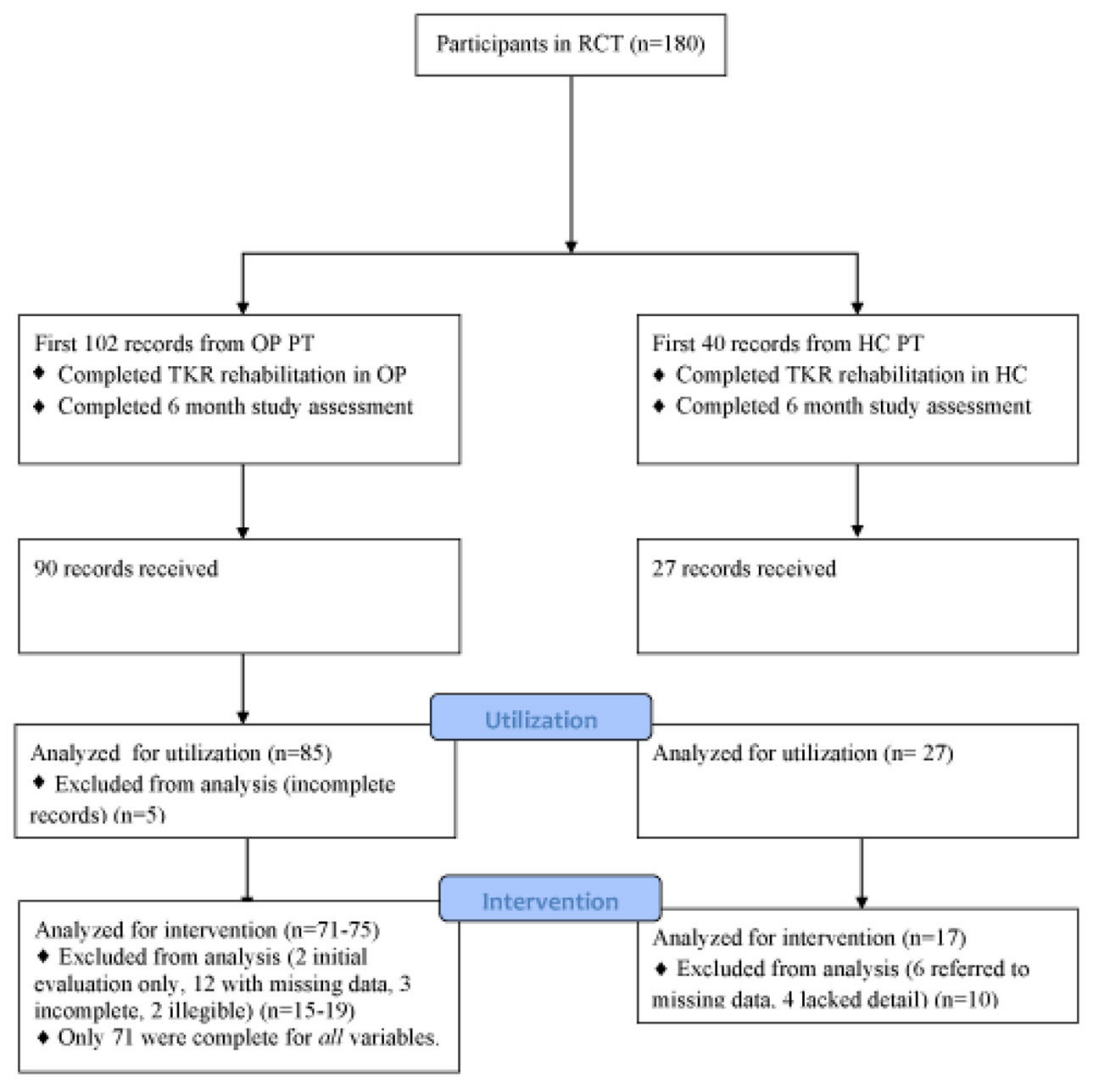
Flow Diagram for PT records

Utilization data: We requested 102 records from 38 outpatient PT facilities and received 90 records (88%) from 32 facilities (Figure 1). These facilities included outpatient physical therapy departments in academic medical centers and community hospitals as well as multi-site and single-site free standing facilities, reflecting the diverse pattern of PT practices in the study region. One facility contributed 22 records. No other facility sent more than 10 records. Two records contained only initial evaluation data, three records were incomplete and one had no discharge information. We requested 40 records from 11 homecare facilities and received 27 homecare records (68%) from 6 facilities. Two facilities contributed 22 records. Baseline characteristics of subjects whose records we received are reported in Table 1 and did not differ from the whole study sample. Individuals who received outpatient PT differed from those who completed their rehabilitation in homecare only in number of co-morbidities. Significantly more individuals in the outpatient group had no co-morbidities (62.2%) compared to those in homecare (37.0%) (p=0.05). Although not statistically significant, a larger percentage (29.6%) of individuals in the homecare group had 2–4 other adults in the home compared to those in the outpatient group (14.4%). It is possible that a larger sample size would reveal a statistically significant difference.

The flow diagram shows the number of records requested, received and analyzed in the homecare and outpatient groups as well as the reasons for missing data.

Pre-MCS: baseline mental component score of the SF-36, scored 0–100, higher scores indicating better mental health; pre-PCS: baseline physical component score of the SF-36, scored 0–100, higher scores indicating better mental health; pre-WOMAC: baseline function subscale of the Western Ontario McMaster Universities Osteoarthritis Index, scored 0–68, lower scores indicating better function; pre-SE: baseline self efficacy score ranging from 0–10, higher scores indicating greater self efficacy; # of co-morbidities: number of painful large joints (hip, knee and spine) in addition to the index knee; # of adults in household: number of adults, including patient, in the household.

Utilization data are reported in Table 2 and demonstrate wide variability of utilization in both the outpatient and homecare settings. Compared to patients who completed their rehabilitation in homecare, patients seen in outpatient PT had more visits (15.6 ± 8.0 visits vs. 10.0 ± 4.6), started and stopped their PT later after surgery (33.6 ± 10.4 days vs. 12.0 ± 3.96 and 97.7 ± 36.1 days vs. 35 ± 10.95 respectively) and stayed in PT longer (65.9 ± 35.0 days vs. 24.0± 10.63) (p<0.001). Additionally 77 (86%) participants in the outpatient group reported also receiving homecare PT prior to outpatient PT; two others could not recall if they had received homecare PT.

Time to initial evaluation in outpatient PT was positively associated with increased number of co-morbidities (p<0.01) and likely associated with BMI (p=0.06) and age (p=0.074). In other words, there was more time between surgery and the beginning of outpatient PT in individuals with more co-morbidities, higher BMI and greater age. Size of insurance co-payments and other baseline characteristics were not significantly associated with utilization variables. Among those who completed their rehabilitation in homecare, BMI was positively y correlated with total number of PT visits (r=0.41; p=0.05) and with days from surgery until discharge from PT (r=0.41; p=0.05). Only three of the 27 individuals in the homecare group provided data about co-payments preventing any analysis of associations between co-payments and PT utilization in this group.

A multiple logistic regression model to predict homecare usage included age, number of adults in the house and baseline MCS score as predictors. Gender was included in the original model but did not contribute significantly to the prediction. Analysis revealed that use of homecare as the terminal rehabilitation site was positively associated with having 2–4 other adults in the home (p=0.05) and marginally associated with older patient age (p=0.08), and poorer mental health (p=0.11). It is noteworthy that participants with 2–4 other adults at home were significantly younger than those alone at home or with one other person (58.8 ± 7.8 vs, 65.6±7.9 years; p=0.001).

Intervention: Of the 90 outpatient records received, 75 (83%) records provided daily notes from which specific exercise interventions could be extracted; 71 records had complete data for all categories. Two records contained only initial evaluations; 12 (13%) records referred to flow sheets not included in the record to detail the reported exercises; three records were incomplete; and two were illegible. Of the twenty-seven records from homecare, 17 (63%) records provided specific exercise interventions. Six records referred to exercise protocols which were not included and four records lacked detail about each treatment session.

All records from both outpatient and homecare settings with intervention data available included exercises to increase knee range of motion. All but two records with available information included some active quadriceps exercise. The two lacking such documentation were from the outpatient setting, one of them stating that the patient discontinued PT following surgeon recommendation when redness of the knee was noted. Four records, all in the outpatient setting, reported the use of neuromuscular electrical stimulation (NMES) to the quadriceps to enhance quadriceps activation.

**Figure 2:**
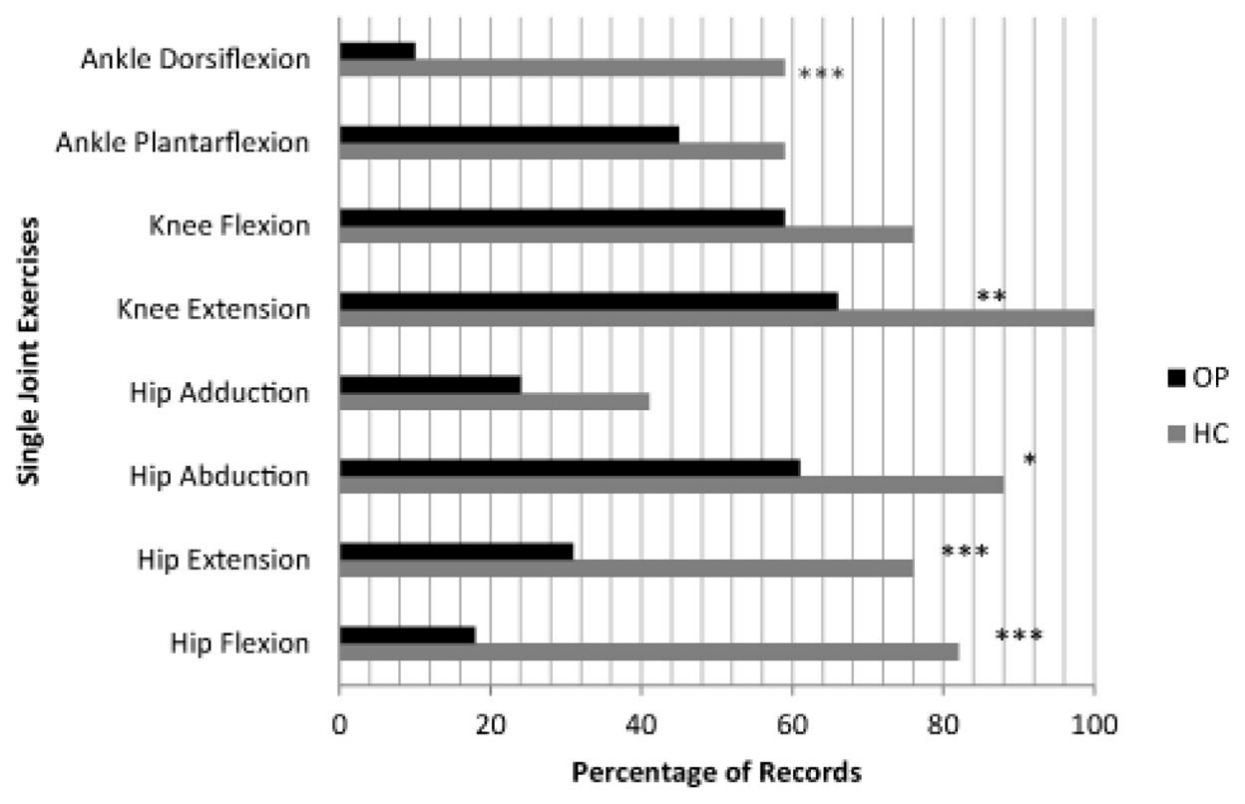
Percentage of records in homecare (gray) and outpatient (black) settings documenting exercises for individual muscle groups The graph reveals differences between home care and outpatient settings’ use of exercises for individual muscle groups as well as variability in groups of muscles exercised. * Indicates statistically significant difference in percentages, based on Proportion test (* p ≤ 0.05, ** p ≤ 0.01, *** p ≤ 0.001).

Although the vast majority of records documented use of some sort of active quadriceps exercise, the mode of exercise varied. Sixty-six percent of records in the outpatient setting indicated use of single-joint quadriceps exercises such as isometric quadriceps setting or knee extension in sitting. All homecare records documented use of single-joint knee extension exercises (Figure 2). Although strengthening for other large muscle groups is recommended in the literature for many lower extremity disorders including TKR, use of specific exercises to strengthen these groups also varied in both settings. For example, over 80% of patients in the outpatient setting performed single-joint hip abduction strengthening exercises but approximately 60% of those in homecare performed hip abduction exercises. In contrast, 60% of homecare patients performed isolated dorsiflexion exercises while only 10% of patients in the outpatient setting did so.

**Figure 3:**
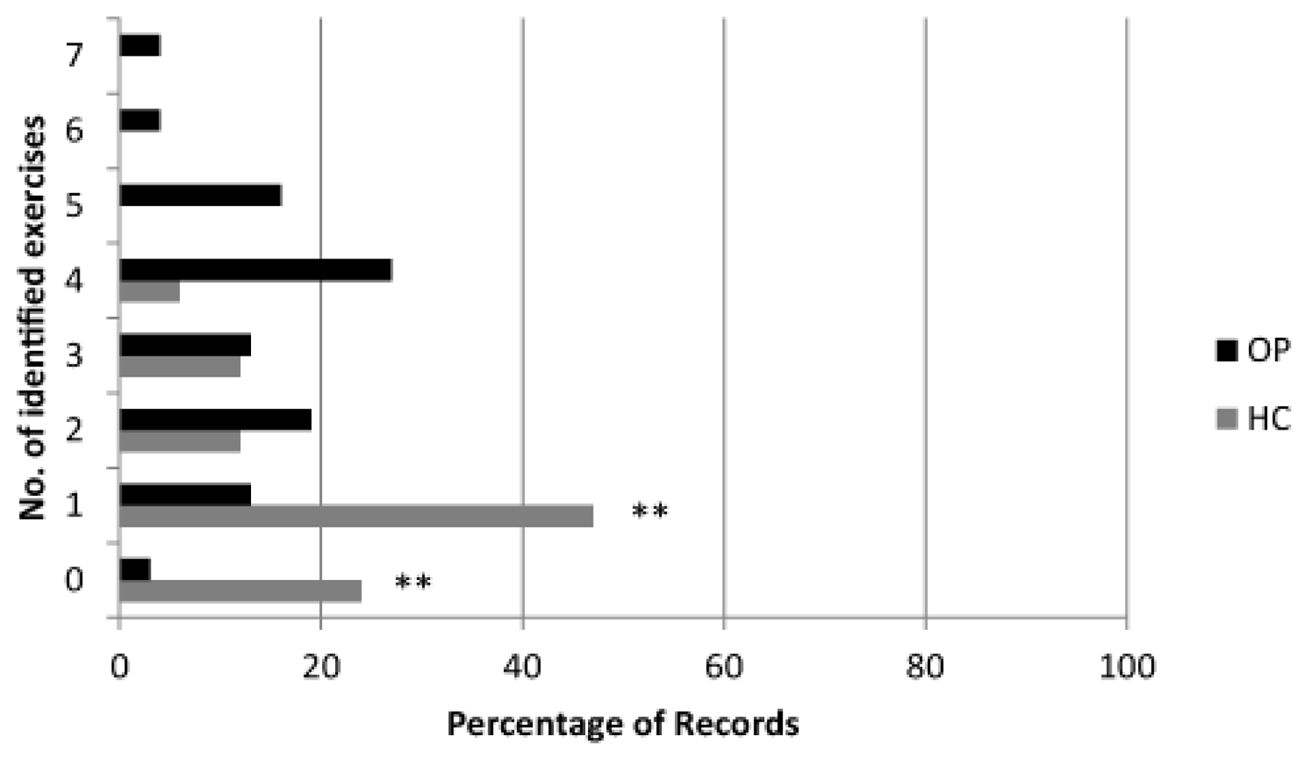
The percentage of records documenting a given number of exercises identified in the literature (homecare in gray, outpatient in black). Comparison of medians revealed a statistically significant difference (p = 0.001) in the median number of identified exercises utilized by the two groups. * Indicates statistically significant difference in percentages, based on Proportion test (* p ≤ 0.05, ** p ≤ 0.01, *** p ≤ 0.001).

**Figure 4:**
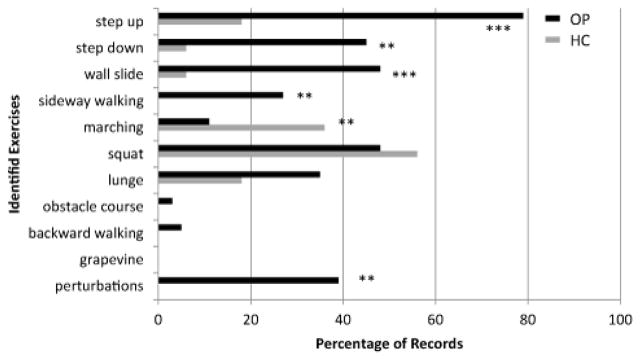
The percentage of records that document the use of specific exercises identified in the literature (homecare in gray, outpatient in black) Comparison of medians revealed statistically significant differences in the use of specific exercises utilized by the two groups. * Indicates statistically significant difference in percentages, based on Proportion test (* p ≤ 0.05, ** p ≤ 0.01, *** p ≤ 0.001).

We identified eleven weight bearing or functional exercises reported in clinical trials or recommended in treatment guidelines in TKR literature (11,12,21). In the 75 outpatient records with available information, all but two records documented use of at least one of these exercises (Figure 3). Thirty-four records (45%) reported using one to three identified exercises and thirty-nine (52%) reported using four or more. The most frequently used exercise in this group was a step-up (Figure 4). Thirteen of seventeen homecare records indicated use of at least one of these identified exercise. Only five homecare records indicated the use of more than one. The most frequently reported weight bearing exercise in homecare was a squat. The median number of identified exercises reported differed significantly between homecare and outpatient settings (1 vs.4, p=0.001).

Sixty-three of the seventy-five outpatient records (84%) documented use of external resistance such as weights. Fourteen of those records documented only one level of resistance. Seventy-two outpatient records had sufficient detail to determine if quadriceps strengthening exercises were progressed. Fifty-four records (60%) documented some level of progression in quadriceps strengthening. In homecare three of seventeen records (18%) indicated use of external resistance and thirteen of seventeen records (76%) documented progression of quadriceps strengthening.

The records provided no standard explanation of reasons for discharge from PT. Some records provided no rationale. Many records noted that “goals were met”. One record noted that insurance benefits had run out and two records reported discharge by physician request. No record provided standardized outcome measures. Similarly complications were not routinely reported.

## Discussion

Although there is growing interest in utilization patterns of PT for a variety of diagnoses, to our knowledge, this is the first study that describes the amount and exercise content of PT administered to patient’s post-TKR. Variation in practice has gained considerable attention in the last decade, as policy makers and healthcare providers seek ways to improve quality of healthcare while maintaining or reducing cost. Practice variation has been described in many healthcare disciplines and is cited as a potential source of excessive healthcare costs and outcome variability [22–24]. Standardization of care and adherence to evidence-based practice in acute care settings following total joint replacements (TJR) resulted in improved short term outcomes and decreased length of hospital stay [25].

Despite current and growing prevalence of TKR surgery, little is known about the optimal amount and composition of rehabilitation services provided to patients following TKR. Westby et al report no consensus among patients, therapists and surgeons on the optimal timing and amount of post-TKR PT [8]. Our data demonstrate considerable variation in timing, amount and exercise content of PT services. The average number of outpatient PT visits seen in our study is consistent with that reported by Fritz [6], particularly in light of data demonstrating that average PT outpatient visit totals are greater in the northeast region of the country where our data were collected [26]. Using a national sample of over 800 patients, DeJong reported an average of 19 home care and/or outpatient PT visits following TKR [5]. Most of our patients who received outpatient PT also received PT in homecare. So the average total PT encounter in our study is likely to be similar to or greater than that reported by DeJong. In contrast those who completed PT in homecare received on average 10 PT visits, approximately half of the total reported by DeJong.

Unlike Freburger [27] who reported increasing age and number of co-morbidities were associated with decreased PT usage, our data showed that number of co-morbidities, and likely older age and greater BMI, were associated with delayed onset of outpatient PT but not with total usage. No other baseline characteristics including mental health status were associated with PT utilization.

The strongest predictor of the use of homecare for the terminal episode of TKR rehabilitation was the presence of two to four other adults in the house in addition to the patient, although there appeared to be a slight positive relationship with older age. An association, albeit weak, between patient age and use of homecare as the terminal site of PT is expected. Older patients are more likely to experience difficulty using outpatient facilities due to limited community ambulation or access to transportation. The presence of two to four other adults in the home as a predictor of homecare as the terminal site may reflect a lifestyle choice in which other family demands limit the ability of a patient to pursue additional physical therapy services in an ambulatory care setting. Further research is needed to fully explain the physical and social factors that dictate patients’ choices of rehabilitation services. Research is also needed to identify optimal rehabilitation strategies that meet the patient’s needs and preferences.

Wide practice variation is also apparent in the content of PT services provided during the terminal rehabilitation experience. The only interventions consistently reported were exercises to restore range of motion and exercises for the quadriceps. Although therapists consistently documented active quadriceps exercises, there was little uniformity in the form or use of resistance or progressive strengthening regimens. Additionally the data suggest that interventions known to be effective in improving functional outcomes 6–12 months post-operatively may be underutilized. Quadriceps strengthening has consistently been shown to improve function following TKR. Despite this, only three-quarters of complete records had any documentation of strengthening progression. Importantly, we conservatively defined progression as any increase in resistance or exercise challenge. No records contained sufficient documentation to determine if progression was adequate to produce muscle strengthening. In many instances, records documented progressions that probably lacked sufficient intensity to promote strengthening. Most but not all records documented use of at least one exercise found in the literature for TKR rehabilitation but the number of identified exercises used varied widely.

The American Physical Therapy Association (APTA) has undertaken several initiatives directed toward enhancing patient care. An APTA committee appointed by the Board of Directors planned meetings to develop strategies to reduce gaps between clinical care and existing research. An underlying assumption made by this committee was that “the quality of physical therapy is threatened by the inappropriate variation in the care delivered” [28]. We are unable to determine from our data whether the variations in PT utilization and content represent sub-optimal or inappropriate patterns. However an understanding of the typical timing of functional recovery reported raises questions about what is the best delivery pattern for PT following TKR. Bade et al show that at one month post-TKR, patients have not returned to pre-operative status in quadriceps strength, knee range of motion or functional performance [29]. At three months post-surgery, these patients had returned to preoperative status in functional performance but continued to exhibit less quadriceps strength than prior to surgery. At six months, these subjects had decreased quadriceps strength and functional performance compared to healthy control subjects. Our data suggest that many patients complete their TKR rehabilitation by one month post-surgery when they are unlikely to have reached their pre-operative status. Perhaps these patients continue their rehabilitation independently by exercising at home or in a gym or community exercise program. Perhaps patients are satisfied with their post-operative status and choose no further care. However Hamilton et al reports approximately 20% of post TKR patients deny overall satisfaction with their outcomes one year post surgery [30]. Nilsdotter et al report 72 percent of subjects reported an expectation of improved sport and recreational activity, but at one year only 25% reported such an improvement [31]. These data suggest that many patients have not achieved their functional goals at one year post-TKR, and perhaps have not achieved their optimal functional level. What is unclear is whether improved function and greater satisfaction would result from more standardized utilization and the application of a less varied and a more intense, evidence-based PT regimen. Similarly it is unknown if outcomes can be improved by optimizing the timing of rehabilitation service delivery. Research to answer these questions should be undertaken to assure optimal physical activity, function and health after TKR.

Data in this study were derived from review of PT records. Not all facilities require the same intervention detail. However we included in the intervention analysis only those records that provided detail. Our system of classifying exercises and documenting progression appears capable of capturing the essential content of a PT encounter. Therefore we expect that those records are an accurate reflection of the PT interventions provided in those episodes. However, virtually none of 117 records from a total of 38 facilities contained enough detail to describe thoroughly the volume and intensity of each individual intervention.

In order to undertake meaningful comparative effectiveness research of rehabilitation practices following TKR, we need a more structured, thorough and precise documentation tool that consistently details the mode of exercise, as well as its frequency, intensity and progression. Similarly documentation must include criteria and rationale for discharge from physical therapy. Without such a system in place, it is impossible to determine the relative effects of intervention, setting, patient characteristics or any other factor on functional outcomes following TKR. The development of such a data capture mechanism is the fundamental step towards comparative effectiveness study of PT care after TKR.

Our results may not be generalizable to other settings. They are derived from a population participating in a clinical trial from a single geographic area of the country. However the subjects whose records we analyzed were similar to the subjects of the entire study. The enrollment rate for the entire sample was over 95% of all eligible subjects so there is little expectation of selection bias. Finally there was no statistical difference in PT utilization between those in the two arms of the clinical trial. Additionally the subjects of the clinical trial are similar to the national population of individuals receiving TKR.

We chose to focus only on the terminal episode of physical therapy care. We recognize that this is only a portion of the rehabilitation care received. However we believe that this is likely to be the most important phase of the rehabilitation program, and a good starting point for this largely understudied issue in PT research. The vast majority of patients following TKR end their formal rehabilitation with either outpatient or homecare services. While a complete understanding of the impact of physical therapy on outcomes following TKR requires an analysis of services provided over the entire spectrum of care, we believe our study is an important first step in determining the elements of physical therapy that produce optimal outcomes.

We had an 88% response rate representing 32 outpatient facilities and believe that intervention data based on 75 records provides important insights into the variability of outpatient PT practice provided to patients following TKR. Although it is unknown if the variability of practice and the apparent under-utilization of interventions known to be beneficial is unique to the geographic area studied, practice variation has been identified throughout the country in other health disciplines. We believe our data reinforce the need for a study of national patterns of physical therapy interventions following TKR. The response rate from the homecare facilities was 67% and two facilities provided the majority of the records. Only seventeen homecare records were complete enough for analysis of the intervention used. We recognize that these homecare data are preliminary and may not represent the state of PT practice in homecare. However we chose to include these data because they are, to our knowledge, the only data available that begin to describe usual PT care in a homecare setting.

Despite these limitations this study is the first that we know of to examine in detail both the quantity and content of PT provided after TKR surgery. The presence of variability in both PT utilization and content complicate any assessment of their roles in determining functional outcomes following TKR. Previous studies report no difference in functional outcomes between patients receiving rehabilitation in the home and those receiving inpatient rehabilitation, outpatient PT or even tele-rehabilitation exercise [32–34]. Our data suggest that such comparisons require considerably more detail about the amount and type of PT services provided in order to determine what, if any, effect the site of rehabilitation or manner of delivery has on functional outcomes. Further research is needed to better understand the sources of practice variation reported in this study and to determine the most appropriate pattern and content of PT services to optimize functional recovery following TKR. Additionally an improved documentation system is needed to advance the study of optimal timing, quantity and content of PT services that will promote the best functional outcomes following TKR.

## Conclusion

There is considerable variation in time to start and end of PT, total number of visits, the kinds of exercises utilized and the use of progressions in rehabilitation post TKR. Current documentation systems lack sufficient detail to fully characterize PT interventions. Explaining and minimizing unwarranted variations in rehabilitation is necessary to determine “best practices” in post TKR rehabilitation.

## Figures and Tables

**Table 1:** Baseline characteristics of subjects whose PT records were reviewed

	Outpatient (n=90)		Homecare (n=27)			All baseline data (n=180)	
	Mean/n	SD/%	Mean/n	SD/%	P-value*	Mean/n	SD/%
Age	63.96	8.44	65.52	7.71	0.432	64.56	8.59
Gender							
Female	57	63.33%	17	62.96%		110	61.11%
Male	33	36.67%	10	37.04%	0.972	70	38.89%
BMI	32.3	5.11	33.69	6.92	0.441	32.48	5.24
pre-MCS	52.76	10.53	49.31	10.91	0.105	51.77	10.93
pre-PCS	33.01	8.46	34.34	7.89	0.474	33.09	8.27
pre-WOMAC	45.29	18.23	42.58	16.34	0.528	45.85	17.27
pre-SE	6.8	2.1	6.49	1.93	0.4	6.72	2.02
# of co-morbidities							
0	56	62.22%	10	37.04%		102	56.67%
1	20	22.22%	12	44.44%		53	29.44%
2	12	13.33%	3	11.11%		19	10.56%
3	2	2.22%	2	7.41%	0.05	5	2.78%
4						1	0.56%
# of adults in household							
1	17	18.89%	3	11.11%		34	18.89%
2	57	63.33%	16	59.26%		112	62.22%
3–5	13	14.44%	8	29.63%	0.185	29	16.11%

* P-values of rank sum test for continuous variable, and chi2 test for categorical variable.

**Table 2:** Utilization data extracted from outpatient and homecare physical therapy records

	Outpatient Mean ± SD (range)	Outpatient Median (interquartile range)	Homecare Mean ± SD (range)	Homecare Median (interquartile range)
Total visits	15.6 ± 8.0 (3–53)	14 (10, 18)	10.0 ± 4.57 (4–21)*	10 (7, 13)
Time to initial evaluation in POD	33.6 ±10.4 (range 5–56)	35.5 (26.5, 41.8)	12 ± 3.96 (4–21)*	12 (10.5, 15)
Time to discharge in POD	97.7 ± 36.1 (range 45–213)	96 (71.8, 111.5)	35 ± 10.95 (22–71)*	31 (28.5, 40)
Days in PT	65.9 ± 35.0 (range 13–200)	62.5 (39.5, 80)	24 ± 10.63 (11–56)*	20 (16.5, 28.5)

* Significantly different from outpatient group means (p<0.001)
